# Bovine leukemia virus proviral load in naturally-infected animals

**DOI:** 10.1186/1742-4690-8-S1-A23

**Published:** 2011-06-06

**Authors:** Irene Alvarez, Gerónimo Gutiérrez, Romina Politzki, Cintia González, Cecilia Martínez, Mariela Gammella, Karina Trono

**Affiliations:** 1 Instituto de Virología Centro de Investigaciones en Ciencias Veterinarias y Agronómicas, INTA, Hurlingham, 1686, Argentina

## 

Due to the high prevalence of Bovine Leukemia Virus (BLV) in argentinean dairy herds, classical control based on the individual level of infection do not constitute a feasible strategy. An approach based on selective segregation of animals according to their peripheral-blood proviral load (PVL) could be a practical alternative.

We conducted this work to get information about the frequency distribution of PVL under natural conditions with the purpose to describe the proportion of animals that could be selected as potential low transmitters.

We analyzed the PVL on 332 cattle from four infected farms with different levels of seroprevalence (Farm A: 30%, Farm B: 66%, Farm C: 87% and Farm D: 92%) together with the correlation of PVL with BLV antibody levels.

In all farms, more than 50% of the seropositive animals showed a proviral load lower than our internal calibrator sample which corresponds to 1 infected cell in 100 non-infected cells according to: Farm A: 78%, Farm B: 79%, Farm C: 55% and Farm D: 79%.

The analysis of antibodies showed that low p24-ELISA reactivity is associated with low PVL in the vast majority of animals.

The present study provides biological data that could contribute with the design of a management program that includes a selective segregation policy for BLV control based on the level of proviral load, which represents a measure of integrated viral genome in host cells and a surrogate marker of the level of BLV transmission under natural conditions.

